# Development and evaluation of an automated report‐based chart checking tool in external beam radiotherapy

**DOI:** 10.1002/acm2.70692

**Published:** 2026-07-07

**Authors:** Oleksii Semeniuk, Andrew J. Wroe

**Affiliations:** ^1^ Department of Medical Physics Warren Alpert Medical School Brown University Providence Rhode Island USA; ^2^ Department of Radiation Oncology Rhode Island Hospital Providence Rhode Island USA; ^3^ Department of Radiation Oncology Baptist Health Miami Cancer Institute Miami Florida USA; ^4^ Department of Radiation Oncology, Herbert Wertheim College of Medicine Florida International University Miami Florida USA

**Keywords:** automated initial chart checking, error detection, FMEA, human error, radiation therapy, treatment planning system

## Abstract

**Background:**

Computerized radiotherapy chart checking tools have revolutionized the initial physics chart review process as they offer verification of large amounts of plan parameters in seconds and allow physicists to concentrate on high‐skill‐level items that are challenging for automation. Both commercialized and in‐house chart check solutions typically rely on access to the patient data within the *live* SQL database of the radiation oncology information system (ROS). This is a complex task potentially posing risks to both database integrity and overall system performance when accessed during clinic operation hours.

**Purpose:**

The aim of this study was to develop and test a chart checking tool based on AURA reports that utilize the *reporting* database to detect and analyze the errors occurring during treatment plan preparation in external beam radiotherapy.

**Methods:**

An Automated AURA Report‐based Chart Checking Tool (AARCCT) was developed using the python‐based programming environment in the RayStation treatment planning system (TPS). Following TG‐275 recommendations, the tool verifies specific physics check items in TPS plan data and Varian's ARIA ROS. The ARIA data was captured leveraging advanced Physics Summary (AURA) reports. The AARCCT was tested on > 600 patients receiving various modalities of treatment, including 3D‐CRT, IMRT and electron treatments. In addition to applying the script to the plans immediately following plan development (i.e., before physics check), it was applied to ∼160 plans already reviewed by medical physicists. The detected errors were assessed with failure mode and effect analysis.

**Results:**

The AARCCT was able to analyze over fifty plan parameters in near to real‐time. Before physics review, ∼48.6% of plans contained at least one error, largely low severity. The error rate was relatively constant throughout the year of testing. After physics checks were completed, AARCCT detected errors in ∼37.1% of physicists checked plans. Both before and after physics check, the most common errors were related to inaccuracies in patient setup imaging (24%), prescription (5.6%), written directive (6.3%), patient shifts (5.4%) and contours (2.6%). The error occurrence rate across the dosimetry team was found to be between ∼22% to 62% with no correlation to dosimetrist's experience. The relative error occurrence rate across the radiation oncologist (RO) team was found to be ∼40%–60%. The higher error rates were observed in ROs who were either recent hires or who had > 20 years of experience. Across the physics team, the error occurrence rate was ∼22%–47% with higher rates among those with less than three or more than twenty years of experience.

**Conclusion:**

The AARCCT was found to be an essential tool to reduce error propagation following manual physics plan checks. The automated nature and omission of live ROS database access uniquely allows for smooth integration of the developed tool into the clinical workflow while minimizing the impact to ROS speed, functionality and security. The tool could also be used for evaluation of staff training and establishing uniformity of practice across the radiation therapy team members to improve quality and operational efficiency.

## 1 INTRODUCTION

Quality assurance (QA) plays a critical role in patient safety during radiation therapy with professional bodies, vendors and clinics pursuing multiple pathways in order to minimize potential errors and maximize treatment safety. Given the majority of errors in radiotherapy originate in treatment planning, the initial pretreatment chart check (ICC) performed by medical physicists was found to be the most effective QA step in the radiotherapy workflow.^1^ Task Group (TG) reports 275 and 315 (Medical Physics Practice Guideline 11.a) were released to guide the medical physicist during an initial chart review with 135 and 44 physics check items respectively.^2^, ^3^ As radiotherapy moves towards more complex treatment delivery techniques, such as intensity‐modulated radiotherapy (IMRT) and volumetric‐modulated arc therapy (VMAT), the scope and complexity of treatment plan QA (i.e., the number of items to be checked) is increasing. The increasing complexity in treatment plans, varying levels of staff training/experience, reduced staffing and limited time to complete review tasks—all contribute to increasing risk of treatment error development and propagation.

Treatment safety can be greatly improved if the physics plan check process is aided with automation and computerization. Indeed, since the physics plan check requires cross‐checking of large amounts of similar plan parameters for each patient between the treatment planning system (TPS) and Radiation Oncology Systems (ROS), this process is an ideal candidate for support via automation. The time saved from automatically verifying binary items (for instance, machine parameter propagation), allows the physicist to concentrate on high‐skill‐level items, that are challenging for computerization (evaluation of image fusion, contour validity, treatment plan quality and verification of patient records from various data sources, etc.). In addition, an automated check tool allows a clinical practice to standardize the chart review process, improve quality by removing inter‐reviewer variability, increase compliance with departmental policies and increase the center's throughput by eliminating /reducing planning‐related patient treatment delays.^4^, ^5^, ^6^, ^7^, ^8^, ^9^, ^10^, ^11^

A number of commercial products are currently available, including SunNuclear (SunCheck), LAP (Radcalc, DosimetryCheck), RadFormation (ClearCheck, ClearCalc), that offer automated verification of various parts of the ICC process.^12^, ^13^, ^14^ Despite these advancements, manual checks still prevail in most radiation oncology departments.^10^, ^15^ One reason for this is that commercial solutions are often “a black box” and offer limited customization/control to the end user. Furthermore, acquiring this software might not always be feasible for all clinics due to financial constraints. Lastly, the interface of such solutions with the TPS and ROS can be cumbersome and requires significant support and oversight from IT support services.^6^, ^10^, ^15^ This has motivated researchers to develop in‐house software tailored to a clinic's specific workflow.^4^, ^5^, ^6^, ^7^, ^8^, ^9^, ^10^, ^11^, ^15^, ^16^ Solutions developed within the clinic typically rely on the access to the patient data within the *live* SQL database of the ROS. This is a complex task posing risks to both database integrity and overall system performance when accessed during clinic operation hours. Frequently, to maintain the patient database integrity, access is limited to times outside of clinic hours and to specific users, thus limiting the usefulness of this approach.

This work presents an automated chart review tool that leverages Physics Summary reports from Varian ARIA's AURA *reporting database*, thus alleviating the risk of patient information corruption and/or ROS system overload. These AURA reports are readily available in XLSX format and customizable to particular clinic's needs. In this work, the generic physics summary report has been expanded upon to capture comprehensive data on radiotherapy treatment preparation, following recommendations of TG‐275 and TG‐315. In order to establish the kinetics of error propagation and efficiency of manual physics chart review, the developed ICC tool was applied before and after physics check. The obtained data was analyzed through failure mode and effects analysis (FMEA) and fault tree frameworks in order to establish the significance and root cause of the detected errors. Finally, the executable steps for operational improvement based on the findings are discussed.

## 2 METHODS AND MATERIALS

The Automated AURA Report based Chart Checking Tool (AARCCT) was developed in the RayStation 2023B (RaySearch Laboratories, Stockholm, Sweden) TPS using its Python‐based scripting environment. The script compares the plan data in the TPS against that in ARIA v17 ROS, captured via the AURA report in XLSX format. While ARIA allows saving AURA reports in various formats, XLSX format was chosen since it offers a clear column/row structure that can be readily processed with Python libraries in the RayStation TPS.

The AURA report sources treatment information from the ARIA Data Warehouse/Reporting database. By default, the reporting database updates every thirty minutes (adjustable by the user), which is adequate for routine chart checking purposes. Furthermore, in the case of urgent plan check, there is an option to instantaneously capture live data. A number of AURA report templates are readily available within ARIA, with physics summary report containing much of data relevant to physics plan check including data related to plan parameters (collimator, gantry, couch angles, MU, etc). In this work, we have enhanced the physics summary AURA report to capture additional treatment‐related information recommended in TG‐275 and TG‐315 which was not available in the generic report. Table 1 shows comparison of the treatment data available in the generic and advanced AURA physics summary reports as well as the verification mode for every check item. Physics items were checked in one of three modes, namely: automated (“A”), visualization (“V”) and manual (“M”). The A‐mode items are checked by the AARCCT, while V‐mode items were automatically extracted from the input data and structured in the report for improved visualization upon manual verification. M‐mode check items are to be manually verified by the user within the TPS and/or ROS systems. It should be noted that many of M‐ and V‐ item checks are envisioned to be automated completely in future especially with practice standardization (see Discussion section). The AURA reports are currently manually generated but will be autogenerated in future as well.

**TABLE 1:** Comparison of physics check items available in the generic and advanced AURA report. “A”, “V” and “M” stand for automated check, enhanced visualization and manual verification, respectively.

	Physics check item	Generic	Advanced	Verification method
**TPS vs. ROS data comparison**	Patient demographics			
	Patient name	✓	✓	A
	Patient date of birth		✓	A
	Patient MRN	✓	✓	A
	Hospital	✓	✓	A
	Plan data			
	Field parameters (treatment unit, field ID, energy, jaw positions, gantry/arc angles, collimator angles, MU, tolerance table)	✓	✓	A
	SSD		✓	A
	FFF beam		✓	A
	Nomenclature (field, beam, plan name)	✓	✓	A
	Beam modifiers (wedge, bolus, applicator)		✓	A
	Planning imaging		✓	A
	Prescription			
	Prescription status		✓	A
	Prescription dose	✓	✓	A
	Fractionation	✓	✓	A
	Treatment frequency	✓	✓	A
	Prescription structure(s) and corresponding dose levels		✓	A
	Linked treatment plan	✓	✓	A
	Beam quality	✓	✓	A
	Treatment technique		✓	A
	Mode		✓	A
	Pre‐treatment imaging (modality & number of sessions scheduled)		✓	A
	Reference point(s)	✓	✓	A
	Clinical goals	✓	✓	A
	Notes		✓	A/M
	Patient setup		✓	V/M
	Special instructions (pacemaker, pregnancy)		✓	A
	MLC parameters			M
**ROS‐specific**	21iX timer		✓	A
	Gated treatment		✓	A/V
	PSQA status		✓	A
	Written directive			M
**TPS‐specific**	Examination grid	✓	✓	A
	Dose grid	✓	✓	A
	Dmax value	✓	✓	A
	Dmax in target	✓	✓	A
	Conformity index (CI) & Paddick CI	✓	✓	A
	HU‐to‐density table	✓	✓	A
	Treatment couch model	✓	✓	A
	Dose calculation algorithm	✓	✓	A
	MU per control point per field	✓	✓	A
	Contour parameters (density override, gaps, discontinuity, couch)	✓	✓	A/M
**Other**	Correct scan used for planning			M
	Image registration quality			M
	Dose distribution			A/M
	Contour quality			M
	Plan reflects the intent (target, laterality, etc)			M
	Treatment history			M
	Collision check			M
	TPS plan report			M

Figure S1A of Appendix A shows the AARCCT script interface. It allows user to select the chart to be checked as well as site and prescription data. The clinical goals in the current plan are compared against the site‐/ fractionation‐ specific clinical goal template corresponding to the selected site/prescription. Once the plan check is complete, AARCCT report is automatically generated and saved in the preferred directory. Figure S1B of Appendix A shows a sample AARCCT report. The functionality of the AARCCT can be split into three main analysis categories. Firstly, the patient information, treatment plan congruence to the prescription / directive written by physician as well as machine parameter propagation from TPS to ROS is verified. This is accomplished by comparing TPS data versus that in advanced physics summary report. The second category (ROS‐specific) evaluates the treatment setup accuracy in the ROS (i.e., scheduling, PSQA, etc.). The third category includes TPS‐specific tests such as reviewing for discontinuities in the contours or dose grid size compared to institutional standards. The complete list of items being checked in each of the three sub‐categories is listed in Table 1.

The AARCCT takes approximately 10 seconds to run and generates a PDF report for review and upload to the ROS. The verification status of each item is color‐coded for clear visualization by the user (green for pass and red for fail). The summary also contains a *Plan Info* section used to alert the user about specifics of the given plan that were not automatically verified but are key items to review per institutional guidelines and best practice. For instance, in the case of a deep‐inspiration‐breath hold (DIBH) treatment, the report prompts the physicist to verify the import/quality of the breathing trace and associated thresholds. The report generates date/time as well as captures the initials of the supervising physician, planner and physicist. Finally, the AARCCT contains the time stamps of when the AURA report was generated and when AARCCT was run. This is especially important for centers with mixed TPS/ROS combinations where plans are not being locked by default upon export to ROS or delivery of first fraction. This time record could serve as a reference should the plan (in TPS) and/or prescription (in ROS) be modified.

The AARCCT was tested on two batches of plans: plans rolled out by dosimetry for physics review and plans that have already been checked and treatment approved by medical physicists. The pre‐physics check batch contained 463 plans collected over a year (∼20% of all clinical plans). Breakdown by modality was: 223 VMAT, 2 conformal ARC, 207 3D‐CRT, 24 electron and 7 electron clinical setup plans. Once the tool was run and errors were detected, the plan was pushed back to the dosimetry for further improvement. Upon the plan correction and resubmission, the tool was run again. This process was repeated until all errors were cleared (should more than one iteration be needed).

The post‐physics‐check batch (i.e. already approved and on‐treatment) contained another 159 plans (∼70% of all plans checked by physics over 90‐day period) with 117 VMAT and 42 3D‐CRT plans. All plans were assessed through FMEA framework.^17^ In order to eliminate inter‐observer variability, the detected errors were matched with the values for occurrence rate, severity and relative priority numbers (RPN) taken from Tables S1.A.i., S1.A.ii and S2.A.i. in TG‐275.^2^

## 3 RESULTS

### 3.1 Error occurrence before physics check

**FIGURE 1:**
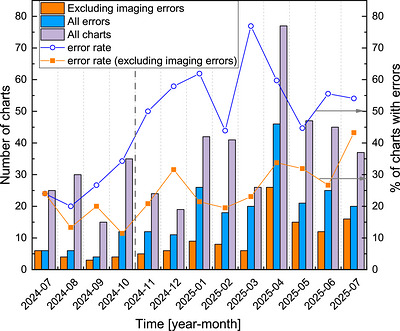
Error occurrence rate over a one‐year term. The vertical dashed gray line indicates onset of imaging modality check with AARCCT.

Figure 1 shows the number of charts checked with the corresponding error occurrence rate by AARCCT over a one‐year term of its development and validation. Of note, the total number of charts released for physics review increased from ∼25 ± 10/month in the second part of 2024 to ∼40 ± 15/month attributed to increased radiation oncologist (RO) staffing. In October 2024, automated verification and tracking of imaging‐related‐errors (i.e., scheduled imaging reflects prescription) was added to the AARCCT. As a result, the error occurrence rate increased from ∼25 ± 5% to ∼55 ± 10%. When imaging‐related errors were excluded from the analysis, the error occurrence rate increased only marginally by ∼5% in the first part of 2025.

**FIGURE 2:**
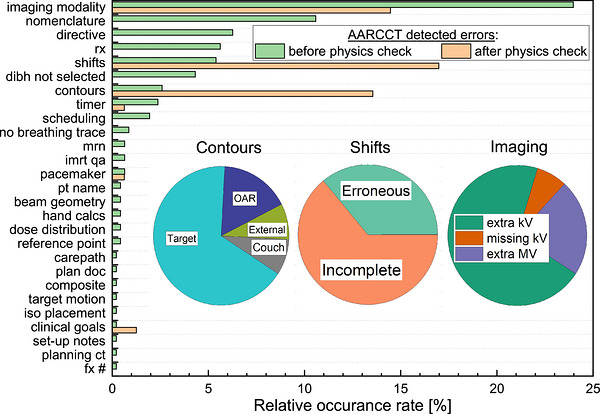
The relative error occurrence rate of with respect to the total number of charts both initially checked following plan generation and after a manual physics check.

Figure 2 shows the relative occurrence rate of a given errors with respect to all errors detected by AARCCT at various chart review stages. It is important to note that some charts contained multiple errors with the error rate displayed relative to the total number of charts checked. Both before physics plan check and after a non‐AARCCT assisted (i.e. manual) physics plan check, the most common errors were related to inaccuracies in patient setup imaging, prescription, written directive (not checked for post‐physics review plans), patient shifts and contours. *Contour errors* detected by the AARCCT tool included discontinuous target, discontinuous organ at risk (OAR) and missing couch contour that constituted 46% and 7.7% and 7.7% of all contour errors, respectively. Other target and OAR‐related contour errors as well as inaccurate external contours were detected manually and constituted 23%, 7.7% and 7.7%, respectively. Among the *directive‐related* errors, 72%, 18% and 9.3% of errors were due to missing notes on prior radiation treatment(s), planning target volume expansions and pacemaker omission, respectively. In 53.9% of *prescription‐related* errors, plans were advanced for physics review before prescription approval by physician was obtained (prescription was in a draft state). Other prescription‐related errors included 15.4% erroneous prescription parameters (dose/fractionation regiment, beam quality), 11.5% missing/erroneous gating, and 3.9% missing imaging and fiducial tracking instructions. Most of the *nomenclature errors* were field (69.4%) and plan (28.5%) name related errors. 64% of *shifts* contained incomplete instructions (for instance, missing anterior/posterior shifts data), while 36% of shifts were erroneous (i.e., wrong values/shift directions). Most of *scheduling errors* were related to scheduling patient‐specific IMRT QA, imaging and treatment sessions. Among the *imaging errors*, 39.3% were due to CBCT and kV imaging being scheduled while only CBCT was prescribed (mostly lung and abdomen VMAT cases); 31.3% were due to MV and kV being scheduled while only kV imaging was prescribed (typically in 3D‐CRT limb and spine treatments); 22.3% were due to MV and kV being scheduled while only MV imaging was prescribed (typically in 3D‐CRT breast and chest wall treatments); and 7.1% were due to both kV and CBCT being prescribed but only CBCT scheduled (typically in head and neck cases). In most timer‐related cases (11), the treatment time limit was too short to complete the field dose delivery while in one case it was much longer than required. Five replans were ordered based errors in isocenter placement, planning fraction number, beam geometry and contours. Further details on errors’ occurrence rate, severity and RPN are given in Table S2 (Appendix B).

#### 3.1.1 Error occurrence across dosimetry staff

**FIGURE 3:**
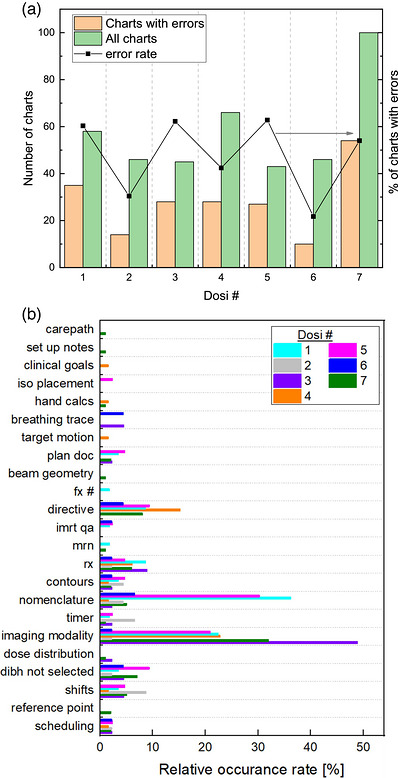
a) Number of charts checked per dosimetry team member, the number of charts with errors and the error rate (black line) displayed. b) The relative occurrence rate of specific error type with respect to the total number of charts produced by each planner.

Figure 3a displays the number of charts per dosimetry team member over a twelve‐month period that was used for the AARCCT performance investigation. As seen from Figure 3a, an average of 56 ± 10 charts were checked per dosimetrist. The exception was Dosi #7 who produced almost double the average number of charts due to the fact that Dosi #7 was planning only 3D‐CRT, electron and hand calculation‐based radiation treatments. Frequently, these treatments were planned and delivered the same day and, in general, required significantly less time than more complex VMAT counterparts. Overall, the error detection rate ranged between 22% to 62% (see Figure 3a) and didn't depend on dosimetrist's experience which was from three to more than 30 years. The chi‐squared test indicated the variation of the error occurrence rate across the dosimetry team was statistically significant (i.e. variation is not due to random sampling) with *p* ≈ 2 × 10^−5^.

The occurrence rate of specific particular error was not uniform across the dosimetry team. For instance, as shown in Figure 3b, AARCCT report analysis showed that Dosi #3 had imaging modality errors (such as prescribed CBCT imaging but scheduled both kV and CBCT and vice versa) in 48.9% of produced plans. For comparison, Dosi #6 only had 2.2% charts with imaging errors. Dosi #1 and #5 had nomenclature errors (particularly beamset name) in 36.2% and 30.2% of charts which is significantly larger than the rest of the dosimetry team, ranging between 1.5–6.5%. Upon further investigation, it was found that Dosi #1 and #5 were consistent in their erroneous beamset names indicating improved understanding of department standards was needed.

#### 3.1.2 Error occurrence across the radiation oncologist staff

**FIGURE 4:**
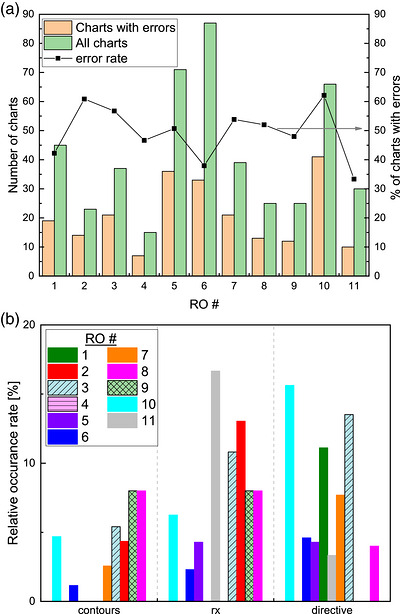
a) Number of charts checked per RO team member, the number of charts with errors and the error rate (black line) displayed; b) The relative error occurrence rate of specific type with respect to the total number of charts per supervising RO.

Figure 4a shows the number of charts checked by AARCCT and the corresponding error occurrence rate for the supervising physicians over a twelve‐month period. For the analysis of error occurrence, only errors related to contours, prescription and directive were considered. The number of charts seen by AARCCT from each RO was between 15 and 87 charts/RO. This was due to a combination of factors, including changes in RO staffing, type of practice (i.e., external beam radiotherapy (EBRT) or brachytherapy service), conferences/holidays as well as the assignment (dosimetry vs on‐call clinical shift) of physics staff involved in AARCCT‐enhanced chart checking. Overall, the relative error occurrence rate was relatively uniform across the RO team, remaining within ∼40%–60% and was not related to years of experience post training. The chi‐squared test indicated that observed error rate variations across the RO staff were not statistically significant (i.e. all ROs have same performance and observed variations are due to random sampling) with *p* ≈ 0.12. However, even though not statistically significant, from the operational standpoint, ROs #2, #3, #10 exhibited error rates on the higher end of spectrum (60.9%, 56.8% and 62.1%, respectively). Interestingly, RO #2, #3 and #10 either had decades of experience or were recent hires with under ten years of experience. RO # 11 also had decades of experience and was on the lower end of spectrum having ∼33.3% of charts with various treatment‐related errors. Interestingly though, when compared with the rest of the RO team, RO #11 had the highest fraction of charts containing more than one error (prescription, imaging, etc). As was observed for dosimetry, some ROs were prone to specific errors. For instance, prescription errors were detected by AARCCT in ∼16 % of RO #11 supervised treatments (particularly in VMAT plans) while most of directive‐relate errors are encountered in RO #3 (15.6%) and RO #10 (13.5%) supervised treatments (see Figure 4b).

### 3.2 Error occurrence after manual physics check

**FIGURE 5:**
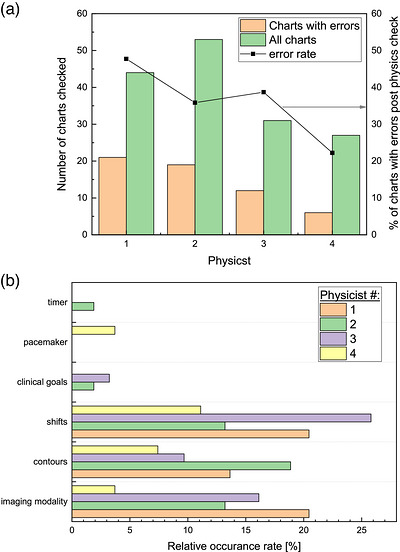
a) Number of charts checked by AARCCT following manual physics check per physics team member. The number of charts with errors and the error rate (black line) are also displayed b) The relative error occurrence rate of specific type with respect to the total number of charts checked by a physicist.

Figure 5a shows the errors detected by AARCCT after manual physics check over three months of testing. The AARCCT checked between 27 to 53 charts per physicist which was dependent on dosimetrist/RO workload as well as the number of chart‐checking shifts per given physicist. The chi‐squared test showed no statistically significant variation of error rates across the physicists with *p* ≈ 0.19. It should be noted that the physics cohort had the smallest sample size, which is believed to limit the reliability and interpretability of the statistical analysis. However, from an operational perspective, it is notable that physicists #1, #2 and #3 (those with either less than three years or more than twenty years of experience) exhibited higher error rates (∼35–47%) compared with physicist #4 (∼10 years of experience), who had an error rate of approximately 27%.

Figure 5b shows the distribution of errors per physicist found by AARCCT. Discontinuous and targets OARs constituted 65% and 35%, respectively of all contour related errors. Among the physics team, the Physicist #3 had most shift errors (25.8%) while contour related errors were highest with Physicist #2 (18.9%). The imaging modality related errors (20.5%) were highest for Physicist #1 who also had the most charts containing errors post physics check at nearly 50%.

## 4 DISCUSSION

This study presents the development of an automated tool for error detection during EBRT preparation, and its evaluation with treatment plans rolled out by dosimetry (i.e., before physics check) and those post manual physics check. The major design criteria of the AARCCT were:

- The patient data query must eliminate the risk of corrupting patient data in the ROS;
- To reduce the potential for development and propagation of errors by filtering and condensing the large amount of information from continually evolving platforms/ modalities requiring human assessment into a manageable checklist;
- Automated and provide and unbiased analysis of treatment plans;
- Low cost and customizable to specific needs of the clinic and deployable across many satellite centers. As such it should allow for smooth integration into the treatment plan development and verification workflow within currently used TPS and ROS across the hospital network.

The development of the AARCCT reported here satisfies the above requirements. As shown in Tables S1 and 2 (Appendix B), it automates many of the check items recommended by TG‐275 and TG‐315 that are currently conducted manually. Interestingly, as seen from Figure 2, while manual physics check is effective in detection of many errors (including severe errors i.e., prescription dose, site, fraction number, etc. (see Table S2 in Appendix B)), the contour‐, shift‐, imaging‐ and nomenclature‐related errors still propagated. AARCCT was able to detect errors early in plan development and thereby expediting the overall plan turnover.

For this work, the AARCCT was run exclusively by a physicist either pre‐ or post‐physics check. However, it is envisioned that it will be run by dosimetry, to pre‐check plans for errors, and then independently by a physicist during the plan check phase. Since the script runs in seconds, this could be done without extending the regular chart check development timeline which may span from under an hour to days. The prompt AARCCT run time was found to be particularly useful for adaptive plans (for instance same day sim and treat), that are typically executed under the time crunch—an environment conducive for error development and propagation.

AARCCT could also assist in new staff onboarding as well as help enforce adherence to departmental policies and standards. For example, Dosi #1 and #5 had nomenclature errors (particularly beamset name) in 36.2% and 30.2% of charts, which is significantly larger than the rest of the dosimetry team ranging between 1.5% – 6.5%. Upon further investigation, it was found that Dosi #1 and #5 were consistent in their erroneous beamset names indicating improved understanding of department standards was needed. Identification of these routine errors with respect to department standards by the AARCCT can provide staff with feedback to ensure best clinical practices are followed, especially if the tool is available for use both pre and during physics checks. The AARCCT could also be used to verify proficiency for independent work following onboarding in a specific modality or disease site. It should be noted though, that while AARCCT tool is envisioned to be used for regular plan checks, having access to it from the first days of training may be suboptimal. Such an approach might foster a reliance on the script and thus undermine the development of crucial manual skills and hinder the development of a deeper understanding of the underlying planning processes.

Furthermore, AARCCT could also be used to improve the departmental efficiency and patient safety. The regular review of AARCCT reports could help leaders to identify gaps in the current processes and suggest the pathways to improvements. Thus, in our analysis, it was found that the relative fraction of plans with errors before and after physics check are comparable. Indeed, when scaled to the total number of charts in each batch, it was determined that 48.6% plans contained errors before physics review. After a manual physics check, AARCCT detected errors remaining in 37.1% of physics checked plans (Figure 3). The errors of RO‐responsibility items (such contours, prescription, directive, etc.) constituted 14.9% and 16.9% of plans in the pre‐physics‐ and post‐physics‐check batches, respectively. This might indicate the that standard operating procedures need to be developed/revised in order to harmonize uniformity across the department. The review of team‐wide (dosimetry/physics/RO) and even more granular operator‐specific errors (see Figures 2, 3, 4, 5) might indicate particular training/re‐training needs of both individuals and groups. For instance, as shown in Figure 3a, Dosi #3 produced imaging scheduling errors in 48.9% of developed plans which was significantly higher than the team average (21.6%). Similarly, while AARCCT report analysis didn't detect contour errors in RO #11 supervised plans, the prescription error rate was on the high end of spectrum ∼16%, vs the RO team average of 6.1% (See Figure 4a). This suggests that extra attention and training should be paid to the staff‐specific treatment‐preparation tasks. In order to further investigate a given error, such an analysis could be performed in a more granular fashion and applied per disease site or per modality.

One of the major challenges after institutional policies are established/revised is staff adherence to them across all centers. The unbiased nature of AARCCT‐report‐based department performance analysis is of value in ensuring standards are maintained. Indeed, via removing any potential human prejudices and conflicts of interests, the AARCCT feedback may be better received by staff and should better facilitate their adherence to departmental standards. Reviewing the annual error rate over the course of the year might further indicate and support staffing changes including the hiring of additional staff to minimize burnout. This is especially important around the holidays and conference periods, when the clinic is working under time and staff pressures—factors that ultimately contributes to error development and propagation (see Figure 1).

One of the greatest factors towards AARCCT adaptability in the clinic is believed to be the fact that it runs off the *reporting database*, as opposed to *live* SQL database of ROS employed in other solutions.^5^, ^6^, ^7^, ^8^, ^9^, ^10^, ^11^, ^12^, ^13^, ^14^, ^15^ This alleviates the risk of patient information corruption and/or ROS system performance degradation and does not require IT support services. Here, it was adapted to EBRT treatments with photons and electrons, but AARCCT allows for easy customization to the evolving needs of the clinic, adaptation of new modalities (such as protons). This is largely due to the modular nature of the tool that is built to work with Varian's AURA report framework. The report template is robust and even upon customization maintains the same structure across modalities. One of the limitations of AURA database is that it contains a limited set of elements from the clinical database. For instance, MLC positions are not part of AURA database and their propagation between TPS/ROS systems remains to be a subject of manual verification. It is envisioned that AARCCT capabilities could be further expanded to include automation of physics check items that are currently being checked manually (see Table 1). For instance, QA of contours and image registration could be performed using machine‐learning algorithms.^18^, ^19^, ^20^, ^21^ At the same time, collisions can be detected via development of 3D models of the patient and LINAC as reported elsewhere.^22^, ^23^ This is the focus of future work by the team.

## 5 CONCLUSIONS

The report‐based automated chart checker demonstrated itself to be an essential clinical tool for a number of applications, ranging from initial chart checking to staff training and improvement of clinical efficiency at both large academic institutions and community centers. Utilization of AURA database ensured that the AARCCT was able to be run in real time without database risk or negative performance impact to the ROS. This computerized solution also enhances the adherence to the institutional policies, however, relies heavily on timely tool utilization and compliance with addressing the items requiring attention.

## AUTHOR CONTRIBUTIONS

Oleksii Semeniuk developed a methodology, collected and analyzed the data, wrote the manuscript. Andrew J. Wroe Wroe provided mentorship, project conceptualization, participated in writing, review and editing.

## CONFLICT OF INTEREST STATEMENT

The authors declare that they have no known competing financial interests or personal relationships that could have appeared to influence the work reported in this paper.

## Supporting information




**Supporting Information**: acm270692‐sup‐0001‐SuppMat.docx

**Supporting Information**: acm270692‐sup‐0002‐SuppMat.docx

**Supporting Information**: acm270692‐sup‐0003‐SuppMat.docx
