# Supplementary figures and images for: Development and evaluation of an automated report‐based chart checking tool in external beam radiotherapy
Source: J Appl Clin Med Phys. 2026 Jul 7;27(7):e70692. doi: 10.1002/acm2.70692 (PMC13341639; doi:10.1002/acm2.70692)

**Appendix A. Supplementary data**

| 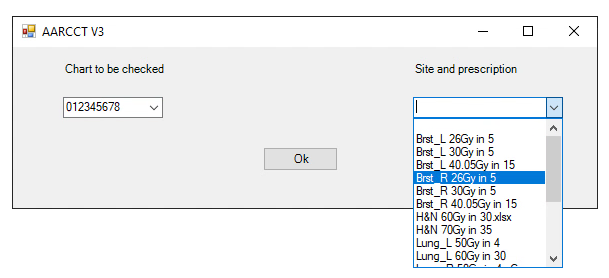  A) |
| --- |
| 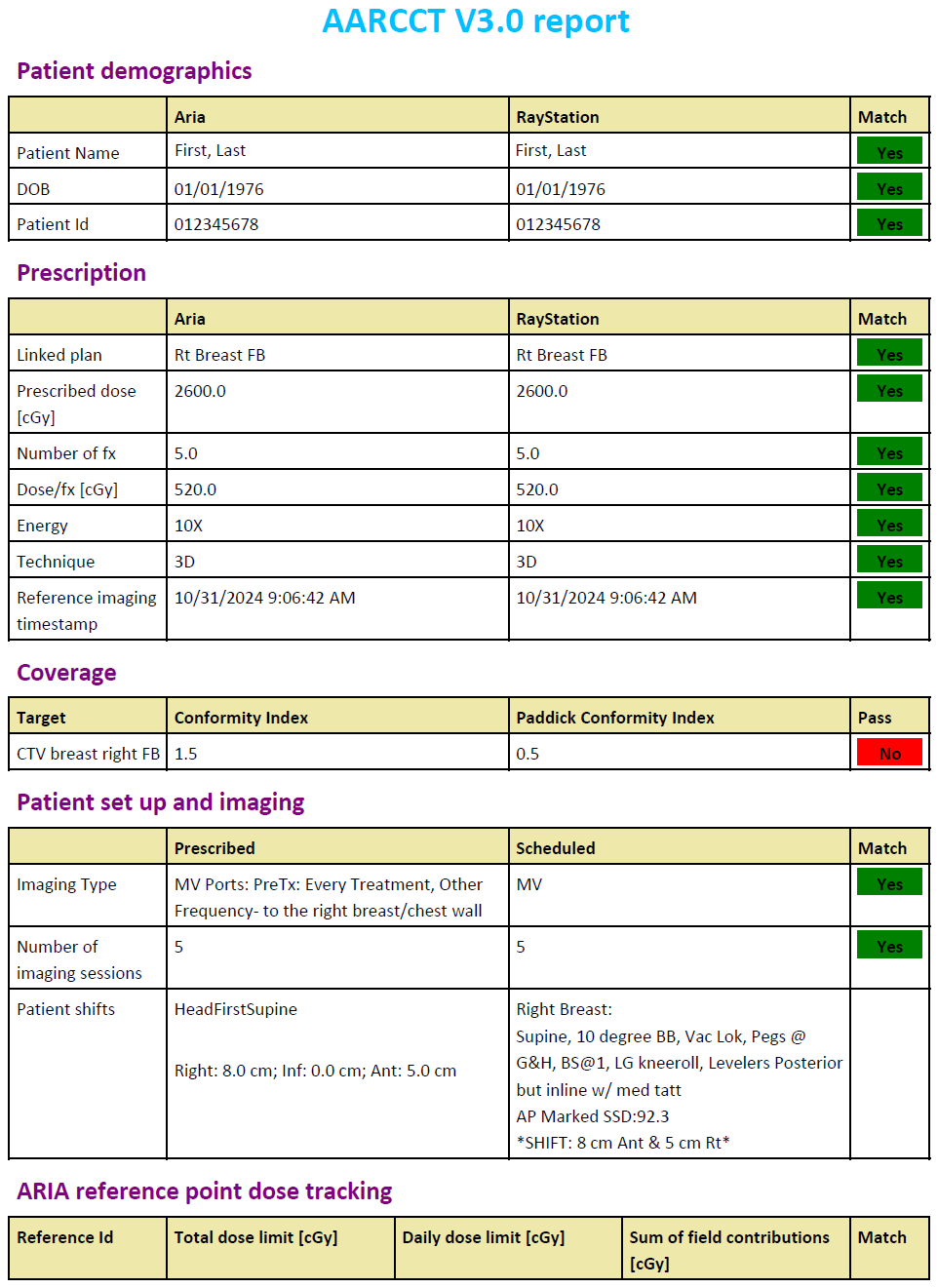  B) |
| 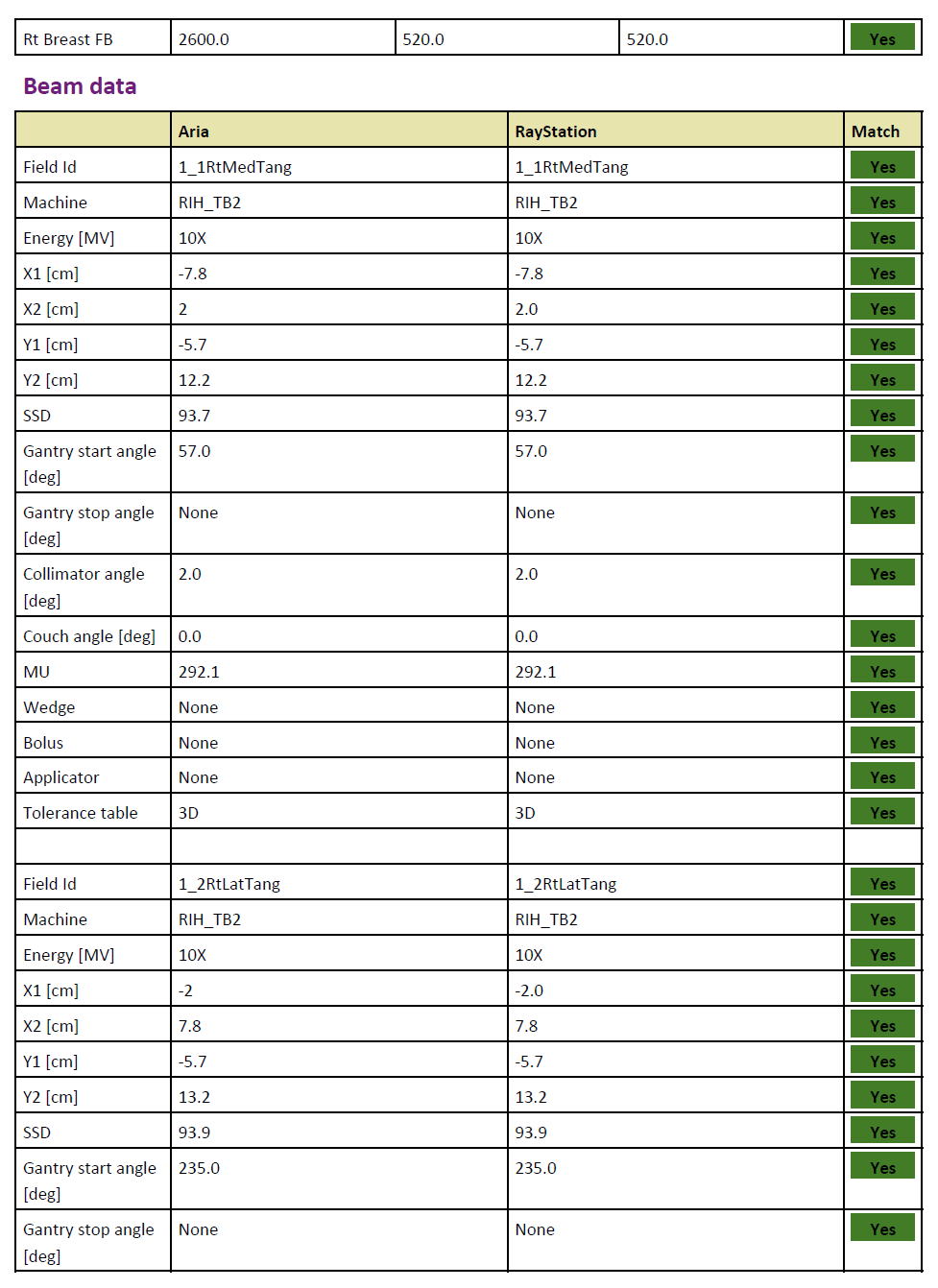 |
| 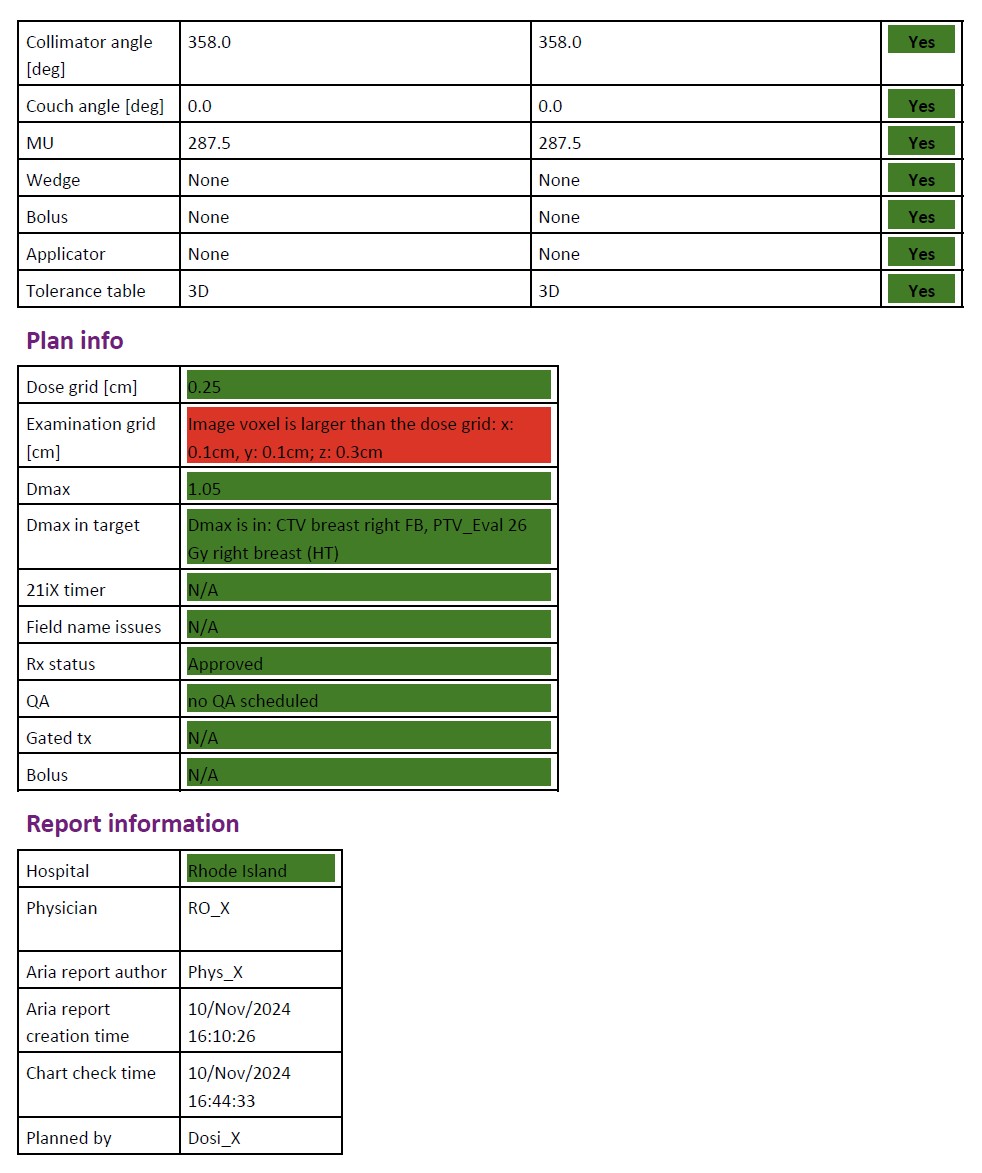 |
| Fig. S-1: A) AARCCT script interface; B) A sample of the AARCCT report. |

Supplement: Supplementary file 1 — Supporting Information: acm270692‐sup‐0001‐SuppMat.docx [file ACM2-27-e70692-s002.docx]
