# Supplementary material for: Development and evaluation of an automated report‐based chart checking tool in external beam radiotherapy
Source: J Appl Clin Med Phys. 2026 Jul 7;27(7):e70692. doi: 10.1002/acm2.70692 (PMC13341639; doi:10.1002/acm2.70692)
Supplement: Supplementary file 2 — Supporting Information: acm270692‐sup‐0002‐SuppMat.docx [file ACM2-27-e70692-s003.docx]

| **Error category** | **Description** | **Number of occurrences** | **Potential risk** | **Severity** | **RPN** | **Corresponding TG-275 item** | **Verification method** |
| --- | --- | --- | --- | --- | --- | --- | --- |
| **Imaging modality** | CBCT is in rx but kV was also scheduled | 44 | Target miss;  OAR overdose; Treatment delay;  Collision issues |  | 144.5  144.5 | TP-Q8-1^2^  TP-Q8-5^2^ | A |
|  | MV is in rx but kV was also scheduled | 24 |  |  |  |  |  |
|  | kV is in rx but MV was also scheduled | 37 |  |  |  |  |  |
|  | kV and CBCT are in Rx but only CBCT was scheduled | 8 |  |  |  |  |  |
|  | kV is in rx but none was scheduled | 1 |  |  |  |  |  |
| **Directive** | Prior Tx record | 23 | Exceeding critical OAR dose tolerances | 7.4 | 214.1  214.1 | 2^1^  PA-Q1-7^2^ | M |
|  | PTV margins | 6 | OAR overdose/ reduced locoregional control | 5.5 | 198  198 | 3^1^  TP-Q1a-4^2^ |  |
|  | Pacemaker | 3 | Transient or permanent device failure | 7.4 | 214.1  214.1 | 2^1^  PA-Q1-6^2^ |  |
|  | Missing directive | 1 | Lack of documentation | 5 | 144.5  175.3 | 13^1^  PA-Q1-1^2^ |  |
| **Rx** | Wrong Rx | 1 | OAR overdose/ toxicities; Reduced locoregional control | 8.2 | 122.5  175.3  175.3 | 26^1^  TP-Q2a-3^2^  TP-Q2a-7^2^ | A |
|  | Dose typo | 1 |  | 6.4 | 175.3  175.3 | 13^1^  TP-Q2a-6^2^ |  |
|  | Draft Rx | 14 |  |  | 175.3  175.3 | PA-Q1-2^2^  TP-Q10-3^2^ |  |
|  | Rx energy differs from TPS | 2 |  | 6.3 | 137.9  144.5 | 17^1^  TP-Q2a-4^2^ |  |
|  | No imaging selected | 3 | Reduced locoregional control due to geographic miss. | 3.6 | 104  180.3  104  261.3 | 45^1^  PA-Q1-3^2^  TP-Q10-6^2^  TP-Q10-8^2^ |  |
|  | Incomplete imaging (ExacTrack not selected) | 1 |  |  |  |  |  |
|  | Rx gating | 4 |  |  |  |  |  |
|  | Fiducials not in Rx | 1 |  |  |  |  | V/M |
| **Contours** | Discontinuous target | 6 | Reduced locoregional control due to an insufficient irradiation field;  Inaccurate dose reporting; | 7.4 | 261.3 261.3 | 3^1^  TP-Q1a-1^2^ | A |
|  | Erroneous target contour | 3 |  |  |  |  | A/M |
|  | Discontinuous OAR | 1 | OAR toxicities;  Inaccurate dose reporting | 7.4 | 261.3 261.3 | 3^1^  TP-Q1a-2^2^ | A |
|  | Erroneous OAR contour | 1 |  |  |  |  | A/M |
|  | External didn’t include immobilization structures* | 1 | Inaccurate dose reporting; Reduced locoregional control due to extra attenuation. | 6.2 | 175.2  261.3 | 7^1^  TP-Q1a-3^2^ | M |
|  | Missing couch* | 1 |  |  | 175.2  261.3 | 7^1^  TP-Q1a-9^2^ | A |
| **Tx preparation**:  Tx scheduling | Not scheduled | 3 | Treatment delay |  | 175.3 | TP-Q2a-7^2^ | A |
|  | Modality | 3 | Inaccurate billing |  | N/A | TP-Q2a-11^2^ |  |
|  | Fx number | 2 | Over/underdose |  | 175.3 | TP-Q2a-7^2^ |  |
| Shifts | Erroneous shifts | 9 | Reduced locoregional control due to geographic miss. | 4.5 | 107.3  107.3  107.3 | 38^1^  TP-Q3a-1^2^  TP-Q6-18^2^ | V/M |
|  | Incomplete shifts | 7 |  |  |  |  |  |
| 21iX timer | Insufficient | 11 | Treatment delay |  | N/A  N/A | TP-Q6-2^2^  TP-Q6-15^2^ | A |
|  | Too large | 1 | Lack of timer-related protection from overexposure |  |  |  |  |
|  | Tx data transfer error | 1 | Over/underdose |  | 107.6  105.1, | TP-Q6-9^2^  TP-Q7a-1 – TP-Q7a-25^2^ |  |
|  | DIBH not selected in ROS | 14 | Reduced locoregional control due to geographic miss;  OAR toxicities |  | 143.2 | TP-Q2a-13^2^ |  |
|  | IMRT QA | 3 | Pt over/under - dose |  | 80 | TP-Q10-10^2^ |  |
|  | No breathing trace | 4 | Pt over/under - dose;  Treatment delay |  | 153.2  104.9 | Sim-Q2-2^2^  Sim-Q2-3^2^ | M |
|  | Set up notes missing | 1 |  | 3.7 | 124.4  124.4 | 24^1^  TP-Q6-6^2^ | V/M |
|  | CarePath was not advanced | 1 | Treatment delay |  |  | PA‐Q1‐12^2^ | M |
| **Treatment planning:**  Nomenclature | Field name | 34 | Wrong field delivered; |  | N/A | TP-Q6-4^2^ | A |
|  | TPS plan name doesn’t match ROS | 14 | Wrong plan delivered |  | 91.3 | TP-Q6-5^2^ |  |
|  | Planning CT name | 1 | Inaccurate dose reporting |  | N/A | Sim-Q2-4^2^ |  |
| Plan quality | Dose distribution (accepted by MD) | 2 | Suboptimal treatment quality; OAR toxicities; Treatment delay |  | 108.3 | TP-Q5-4^2^ | A/V/M |
|  | Excessive target motion (accepted by MD) | 1 |  |  | 153.2 | Sim-Q2-1^2^  TP‐Q1a‐1,2,3,4,7^2^ | M |
|  | Wrong fx #* | 1 |  | 5.4 | 143.2  175.3 | 15^1^  TP-Q2a-7^2^ | A |
| Beam arrangement | Beams through prosthesis (accepted by MD) | 1 | Suboptimal treatment quality; Reduced locoregional control; Sensitivity to pt setup |  | 108.3 | TP-Q6-1^2^ | M |
|  | e-beam was not orthogonal* | 1 | Suboptimal treatment quality Inaccurate dose reporting; |  | 108.3 | TP-Q6-1^2^ | M |
|  | ISO placement* | 1 | Treatment delay;  Failing gantry clearance | 5.5 | 107  N/A  N/A | 39^1^  TP-Q6-2^2^,  TP-Q6-17^2^ | A/M |
|  | Hand calc (replan) | 2 | Over/underdose | 5.2 | 107.6  107.6 | 37^1^  TP-Q6-9^2^ | M |
| Treatment planning evaluation and documentation | Composite missing | 1 | Risk of exceeding OAR dose tolerances; OAR toxicities; | 7 | 150.3 | TP-Q5-7^2^ | M |
|  | Clinical goals | 1 |  |  | 150.3 | TP-Q5-1^2^ | A |
|  | No Rx IDL in plan doc | 1 | Inaccurate dose reporting;  Challenges in replicating the dose distribution in future without electronic records |  | N/A | TP-Q5-8^2^ | M |
|  | CT slice thickness (OK) | 1 | Inaccurate dose reporting; | 4.8 | 122.8  33.4  261.3 | 25^1^  Sim-Q1-10^2^  TP-Q10-2^2^ | A |
| **Pt demographics** | MRN | 3 | Treating wrong patient | 3.1 | 71.6 | 48^3^ | A |
|  | Pt name | 2 |  |  |  |  |  |

Description of errors detected by AARCCT in the plans rolled out by dosimetry over a 12-month period. Data from TG-275 Table: ^1^ – S1.A.i; ^2^ – S1.A.ii; ^3^ – S2.A.i. * – physics check item triggered a replan. Rx for prescription. “A”, “V” and “M” stand for automated check, enhanced visualization and manual verification, respectively.
